# Identification of genetic loci associated with renal dysfunction after lung transplantation using an ethnic-specific single-nucleotide polymorphism array

**DOI:** 10.1038/s41598-023-36143-y

**Published:** 2023-06-01

**Authors:** Yasuaki Tomioka, Seiichiro Sugimoto, Haruchika Yamamoto, Shuta Tomida, Toshio Shiotani, Shin Tanaka, Kazuhiko Shien, Ken Suzawa, Kentaroh Miyoshi, Shinji Otani, Hiromasa Yamamoto, Mikio Okazaki, Masaomi Yamane, Shinichi Toyooka

**Affiliations:** 1 Department of General Thoracic Surgery and Breast and Endocrinological Surgery, Okayama University Graduate School of Medicine, Dentistry and Pharmaceutical Sciences, Okayama, Japan; 2 Department of General Thoracic Surgery and Organ Transplant Center, Okayama University Hospital, 2-5-1 Shikata-cho, Kita-ku, Okayama, 700-8558 Japan; 3 Center for Comprehensive Genomic Medicine, Okayama University Hospital, Okayama, Japan

**Keywords:** Genetic predisposition to disease, Kidney diseases, Respiratory tract diseases, Translational research, Allotransplantation

## Abstract

Renal dysfunction is a long-term complication associated with an increased mortality after lung transplantation (LT). We investigated the association of single-nucleotide polymorphisms (SNPs) with the development of renal dysfunction after LT using a Japanese-specific SNP array. First, eligible samples of 34 LT recipients were genotyped using the SNP array and divided into two groups, according to the presence of homozygous and heterozygous combinations of mutant alleles of the 126 renal-related SNPs. To identify candidate SNPs, the renal function tests were compared between the two groups for each SNP. Next, we investigated the association between the candidate SNPs and the time course of changes of the estimated glomerular filtration rate (eGFR) in the 99 recipients until 10 years after the LT. ΔeGFR was defined as the difference between the postoperative and preoperative eGFR values. Eight SNPs were identified as the candidate SNPs in the 34 recipients. Validation analysis of these 8 candidate SNPs in all the 99 recipients showed that three SNPs, namely, rs10277115, rs4690095, and rs792064, were associated with significant changes of the ΔeGFR. Pre-transplant identification of high-risk patients for the development of renal dysfunction after LT based on the presence of these SNPs might contribute to providing personalized medicine.

## Introduction

Lung transplantation (LT) is an established therapeutic option for selected patients with end-stage pulmonary disease. Although advances in immunosuppressive drug therapy have improved the outcomes after solid organ transplantation, the survival rate after LT has been lower than that after other organ transplantation^1,2^. Life-long immunosuppressive therapy, including with calcineurin inhibitors (CNI), mycophenolate mofetil and glucocorticoids is required after LT to prevent chronic lung allograft dysfunction (CLAD), this latter condition being the main hindrance to long-term survival^3–5^. However, long-term immunosuppression is also associated with the risk of development of chronic kidney disease (CKD), potentially necessitating initiation of chronic dialysis or renal transplantation after LT^6–10^. According to the registry report from the International Society for Heart and Lung Transplantation (ISHLT), severe renal dysfunction, defined by a serum creatinine level of 2.5 mg/dL or higher and the need for chronic dialysis or renal transplantation, is estimated to occur in 5.6% of patients at 1 year, 16.0% of patients at 5 years, and 24.6% of patients at 10 years after LT^1^. Importantly, severe renal dysfunction is associated with increased mortality^7^. Fluid retention caused by CKD has a negative effect on the lung allograft, leading to the development of infection and CLAD. In addition, therapeutic options for late complications after LT, including CLAD, infection, and malignancy, is definitely limited due to poor kidney function in patients with CKD. Therefore, preventing the development of CKD over the long term is necessary to improve the prognosis after LT.

Genome-wide association studies have been performed to identify novel genetic associations for many diseases. Recently, a meta-analysis of genome-wide association studies identified multiple genetic loci associated with kidney function-related traits in east Asian populations^11^. In relation to LT, various kinds of single-nucleotide polymorphisms (SNPs) have been shown to be associated with the rates of utilization of donor lung allografts^12^, risk of development of bronchiolitis obliterans syndrome^13^, risk of development of primary graft dysfunction (PGD)^14^, rate of postoperative decline in the total lung capacity^15^, and infection free-survival after discharge from the intensive care unit in recipients of living-donor lobar LT (LDLLT)^16^. However, the associations between SNPs and the risk of development of renal dysfunction over the long term after LT have not yet been elucidated. Identification of the SNPs might contribute to distinguishing high-risk patients for the development of renal dysfunction and offering personalized medicine to improve prognosis of these patients after LT. The purpose of this study is to explore SNPs associated with the risk of development of renal dysfunction over the long term after LT.

In this study, we investigated the SNPs that might be associated with the risk of development of renal dysfunction over the long term after LT using an ethnic-specific SNP array for the Japanese population, the Japonica Array NEO (Toshiba, Japan), which comprises a total of 66,883 markers^17^.

## Results

### Exploratory cohort analysis

The characteristics of the 34 recipients in the exploratory cohort are shown in Table 1. The patients had normal renal function, with a median eGFR of 95.8 (46.0–174.0) mL/min/1.73 m^2^ prior to LT. The prevalence rates of posttransplant diabetes mellitus^18^, hypertension^18,19^, and dyslipidemia^18^, which are known as risk factors for posttransplant renal dysfunction, were 8.8%, 79.4%, and 26.5%, respectively. Two patients (5.9%) required hemodialysis after LT. The median follow-up period was 8.40 (2.20–21.9) years after LT.

**Table 1:** Patient characteristics in the exploratory cohort and the validation cohort.

Variable	Exploratory cohort (N = 34)	Validation cohort (N = 99)
Preoperative variables		
Age, years, median (range)	40.5 (23.0–59.0)	40.0 (18.0–68.0)
Sex (female), n (%)	22 (64.7%)	59 (59.6%)
Body mass index, kg/m^2^, median (range)	18.5 (11.2–25.2)	18.6 (10.7–29.3)
Diagnoses		
Interstitial lung disease	7 (20.6%)	31 (31.4%)
Emphysema	6 (17.6%)	8 (8.1%)
Pulmonary graft-versus-host disease	6 (17.6%)	13 (13.1%)
Lymphangioleiomyomatosis	6 (17.6%)	13 (13.1%)
Pulmonary hypertension	4 (11.8%)	13 (13.1%)
Bronchiectasis	3 (8.9%)	13 (13.1%)
Other diseases	2 (5.9%)	8 (8.1%)
Lung allocation score, median (range)	37.0 (22.1–88.0)	39.6 (22.1–89.9)
CMV mismatch (recipient negative/donor positive)	2 (5.9%)	8 (8.1%)
Lung donor		
Living donor	7 (20.6%)	34 (34.3%)
Deceased donor	27 (79.4%)	65 (65.7%)
Calcineurin inhibitor		
Tacrolimus	33 (97.1%)	85 (85.9%)
Cyclosporine	1 (2.9%)	14 (14.1%)
Pretransplant creatinine (mg/dL), median (range)	0.58 (0.33–1.21)	0.58 (0.27–1.21)
Pretransplant eGFR (mL/min/1.73 m^2^), median (range)	95.8 (46.0–174.0)	99.0 (46.0–322.6)
Preoperative diabetes mellitus, yes	3 (8.8%)	9 (9.1%)
Preopeartive hypertension, yes	1 (2.9%)	3 (3.0%)
Preoperative dyslipidemia, yes	3 (8.8%)	6 (6.1%)
Intraoperative variables		
Lung transplant procedure		
Single	9 (26.5%)	19 (19.2%)
Bilateral	25 (73.5%)	80 (80.8%)
Operative time (min), median (range)	443.5 (236.0–690.0)	469.0 (219.0–845.0)
Ischemic time (min), median (range)	534.0 (89.0–769.0)	462.0 (82.0–787.0)
Cardiopulmonary bypass use, yes	23 (67.6%)	75 (75.8%)
Postoperative variables		
Maximum grade of PGD (0–72 h), median (range)	1.5 (0–3)	2.0 (0–2)
Acute rejection, yes	10 (29.4%)	37 (37.4%)
Antibody-mediated rejection, yes	2 (5.9%)	10 (10.1%)
Postoperative diabetes mellitus, yes	3 (8.8%)	14 (14.1%)
Postopeartive hypertension, yes	27 (79.4%)	81 (81.8%)
Postoperative dyslipidemia, yes	9 (26.5%)	19 (19.2%)
Hemodialysis after transplant, yes	2 (5.9%)	6 (6.1%)
Time to hemodialysis after transplant (year), median (range)	14.6 (11.5–17.7)	12.3 (4.40–17.7)
Chronic lung allograft dysfunction, yes	7 (20.6%)	23 (23.2%)
Time since transplant to follw-up (year), median (range)	8.40 (2.20–21.9)	7.90 (0.80–23.4)

Data are presented as n, median (range) or n (%). *CMV* cytomegalovirus, *eGFR* estimated glomerular filtration rate, *PGD* primary graft dysfunction.

**Figure 1:**
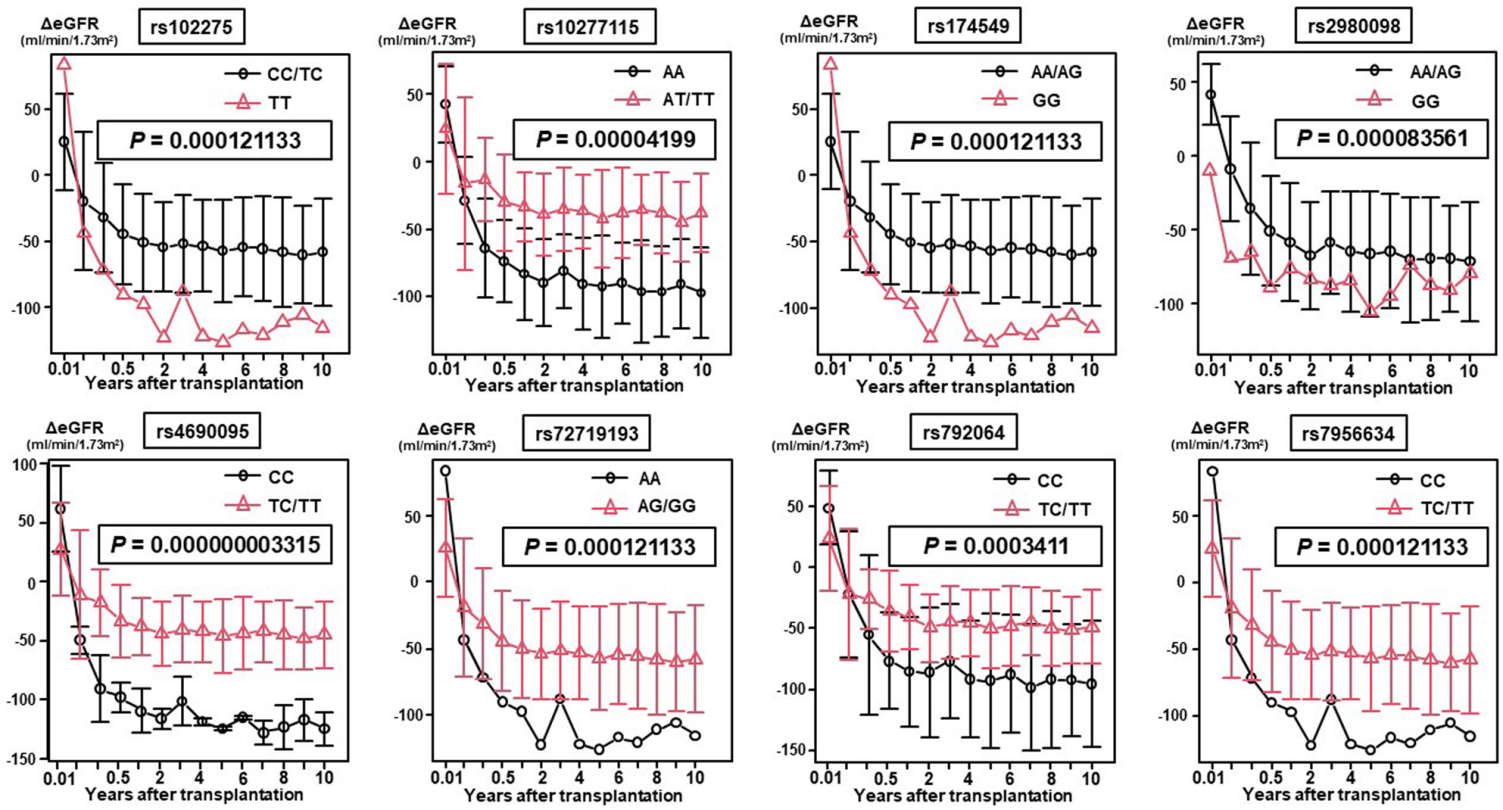
In the exploratory cohort, 8 single-nucleotide polymorphisms (SNPs), including rs102275 (*P* = 1.2 × 10^–4^, MAF = 0.33), rs10277115 (*P* = 4.2 × 10^–5^, MAF = 0.25), rs174549 (*P* = 1.2 × 10^–4^, MAF = 0.33), rs2980098 (*P* = 8.4 × 10^–5^, MAF = 0.33), rs4690095 (*P* = 3.3 × 10^–9^, MAF = 0.47), rs72719193 (*P* = 1.2 × 10^–4^, MAF = 0.49), rs792064 (*P* = 3.4 × 10^–4^, MAF = 0.40), and rs7956634 (*P* = 1.2 × 10^–4^, MAF = 0.34), were identified as candidate SNPs associated with significant differences in the ΔeGFR (postoperative eGFR − preoperative eGFR) during the first 10 years after lung transplantation.

Of the total of 126 renal-related SNPs, 8 SNPs, including rs102275 (*P* = 1.2 × 10^–4^, MAF = 0.33), rs10277115 (*P* = 4.2 × 10^–5^, MAF = 0.25), rs174549 (*P* = 1.2 × 10^–4^, MAF = 0.33), rs2980098 (*P* = 8.4 × 10^–5^, MAF = 0.33), rs4690095 (*P* = 3.3 × 10^–9^, MAF = 0.47), rs72719193 (*P* = 1.2 × 10^–4^, MAF = 0.49), rs792064 (*P* = 3.4 × 10^–4^, MAF = 0.40), and rs7956634 (*P* = 1.2 × 10^–4^, MAF = 0.34), were identified as candidate SNPs associated with significant differences in the postoperative changes of the ΔeGFR over a 10-year period after LT (Fig. 1). Detailed information on the chromosome, reference allele, alternation allele, gene, location, and MAF is presented in Table 2.

**Table 2:** Detailed information of candidate SNPs.

dbSNP	Chromosome	Reference allele	Alternation allele	Locus	Gene symbols	Location	Minor allele frequency	Function	*P* value
rs102275	11	C	T	11q12.2	TMEM258	61557803	0.327	Intronic	1.21 × 10^–4^
rs10277115	7	A	T	7p22.3	UNCX	1251721	0.250	Regulatory	4.20 × 10^–5^
rs174549	11	G	A	11q12.2	FADS1	61803910	0.327	Intronic	1.21 × 10^–4^
rs2980098	4	A	G	4p16.3	TMEM128	4251229	0.332	Intergenic	8.36 × 10^–5^
rs4690095	4	C	T	4p16.3	RGS12	3419582	0.471	Intronic	3.32 × 10^–9^
rs72719193	14	G	A	14q24.1	GPHN, TMEM229B	67502391	0.486	Intronic	1.21 × 10^–4^
rs792064	2	C	T	2p25.2	SOX11	5378545	0.399	Intergenic	3.41 × 10^–4^
rs7956634	12	T	C	12p12.3	RERG	15168260	0.341	Intronic	1.21 × 10^–4^

### Validation cohort analysis

The characteristics of the 99 recipients in the validation cohort are shown in Table 1. The pre-transplant median eGFR value was normal in all the recipients, at 99.0 (46.0–322.6) mL/min/1.73 m^2^. The prevalence rates of posttransplant diabetes mellitus, hypertension, and dyslipidemia were 14.1%, 81.8%, and 19.2%, respectively. Six patients (6.1%) eventually required hemodialysis after LT. The median follow-up period was 7.90 (0.8–23.4) years after LT.

**Figure 2:**
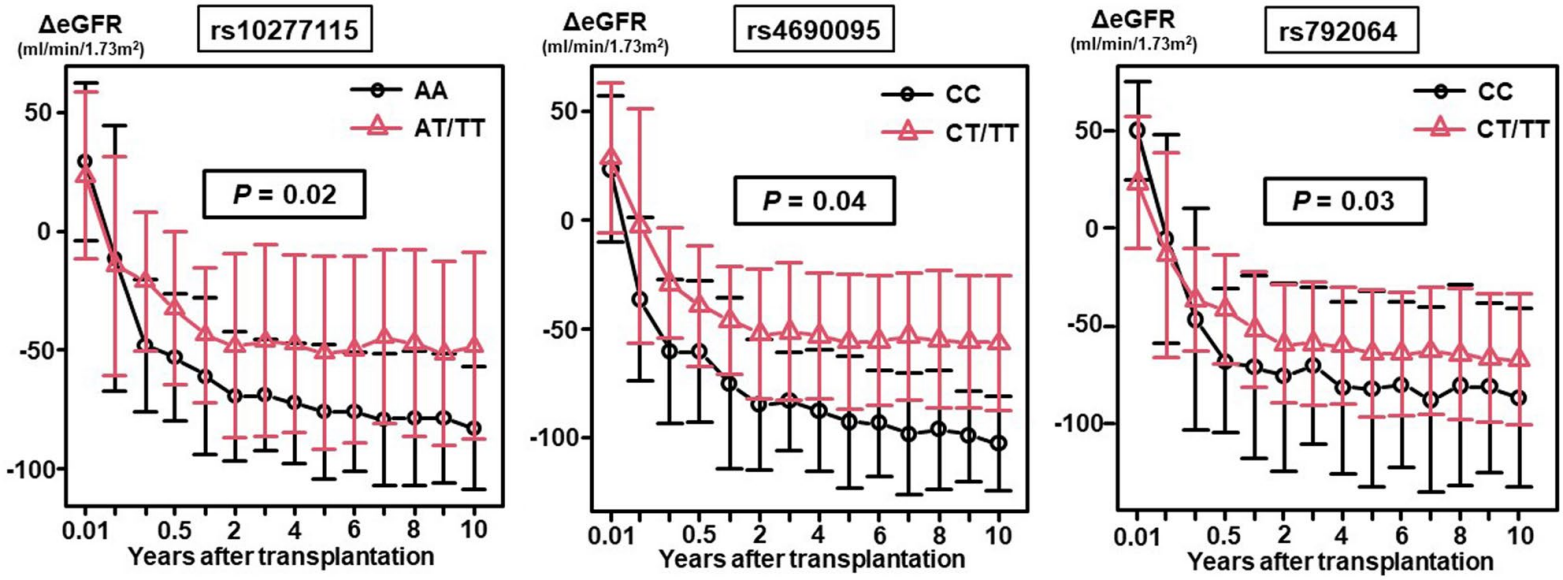
In the validation cohort, rs10277115, rs4690095, and rs792064 among of 8 candidate single-nucleotide polymorphisms (SNPs) were associated with significant differences in the ΔeGFR (postoperative eGFR − preoperative eGFR) during the first 10 years after lung transplantation.

**Figure 3:**
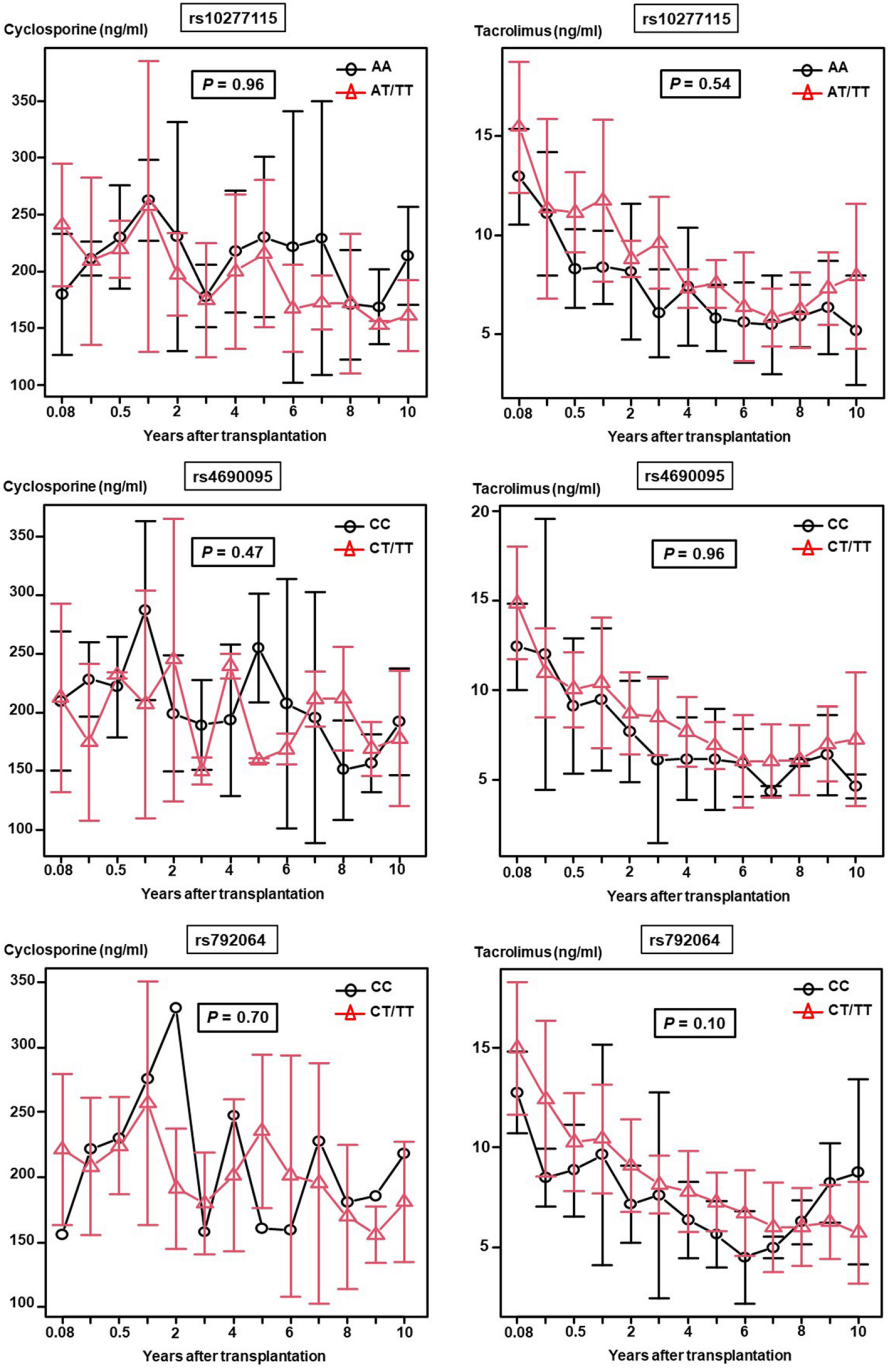
In the validation cohort of 99 recipients, there were no significant differences in the trough level of cyclosporine or tacrolimus during the first 10 years after lung transplantation between the two groups for the three identified single-nucleotide polymorphisms (SNPs) (rs10277115; cyclosporine (*P* = 0.96), tacrolimus (*P* = 0.54); rs4690095, cyclosporine (*P* = 0.47), tacrolimus (*P* = 0.96); rs792064; cyclosporine (*P* = 0.70), tacrolimus (*P* = 0.10)).

Validation analysis of the 8 candidate SNPs showed that three of the SNPs (rs10277115, rs4690095, and rs792064) were associated with significant differences in the postoperative changes of the ΔeGFR over a 10-year period after LT (Fig. 2). The postoperative changes of the ΔeGFR were significantly lower in the AA group for rs10277115, CC group for rs460095, and CC group for rs792064 (*P* = 0.02, 0.04, and 0.03, respectively). Figure 3 shows the mean trough level of CNIs during the first 10 years after LT in the three identified SNPs, and there were no significant differences between the two groups for the three SNPs. The characteristics of the patients with each of the 3 SNPs are shown in the Supplementary data. In the patients with rs10277115, there was a significant difference in the distribution of the underlying disease between the two groups (*P* = 0.034) (Supplementary Table S1). Interestingly, the pretransplant creatinine was significantly lower (*P* = 0.023) and pretransplant eGFR was significantly higher (*P* = 0.008) in the CC group than in the CT/TT group for rs4690095 (Supplementary Table S2). Although the renal function before the LT was worse in the CT/TT group, it was better preserved over time in this group as compared with that in the CC group. The patient characteristics did not differ between the two groups classified according to homozygosity/heterozygosity for rs792064 (Supplementary Table S3).

Additional analysis of the 8 candidate SNPs in the 65 recipients except the 34 recipients in the exploratory cohort showed that rs4690095 was associated with significant difference in the postoperative changes of the ΔeGFR over a 10-year period after LT (*P* = 0.048) (Supplementary Fig. S1). The characteristics of the 65 recipients in the validation cohort are shown in Supplementary Table S4 and the patient characteristics did not differ between the two groups classified according to homozygosity/heterozygosity for rs4690095 (Supplementary Table S5).

## Discussion

In the present study, we found from our analysis using a Japanese-specific SNP array that three SNPs, namely, rs10277115, rs4690095, and rs792064, among the 8 candidate SNPs were associated with significant differences in the postoperative changes of the ΔeGFR over a 10-year period after LT. Presence of these three SNPs was associated with the development of renal dysfunction in the long term after LT, suggesting that these SNPs might represent unique predictive risk factors for the development of CKD over the long term after LT. Furthermore, genotypic assessment for the presence of these SNPs prior to LT may contribute to improvement in personalized medicine, such as provision of individualized immunosuppressive therapy to protect the kidney function over the long term after LT. To the best of our knowledge, this study is the first to explore SNPs associated with the risk of development of renal dysfunction over the long term after LT.

We demonstrated that the T alleles of rs10277115, rs4690095, and rs792064 might be nephroprotective in patients receiving long-term immunosuppressive therapy after LT. The first SNP, rs10277115 in the *UNCX* gene, which encodes a paired-type homeobox transcription factor and has essential roles in skeleton formation and kidney development^20^, has been shown to be associated with an increased risk of development of CKD in East Asian populations, including Japanese^11^. The second SNP, rs4690095, that has been mapped to the *RGS* gene involved in transcription factors and tumorigenesis, which was identified in a genome-wide association analysis conducted using 58 clinical tests in 160,000 Japanese subjects, was reported to be associated with the serum creatinine levels, eGFR values, and serum albumin levels^21^. The third SNP, rs790264, which is located in an intergenic region, was identified from a genome-wide association analysis conducted for a European pediatric CKD cohort as being associated with proteinuria^22^. As previously reported, Asian populations are reported as being at a lower risk of developing CKD after non-renal solid organ transplantation^7^. The risk of CKD after transplantation might be influenced by racial differences represented by SNPs associated with the risk of renal dysfunction developing over the long term after transplantation, as shown in the present study.

The results of this study revealed that the nephroprotective effect of each SNP was marked from 3 to 6 months, reached a plateau between 6 months and 1 year, and was maintained over the long-term after LT. In fact, the decline of renal function after LT is known to be usually biphasic, with a rapid decline in the first post-transplant year and a slower decline thereafter^9^. After LT, acute kidney injury in the perioperative period results from the administration of multiple drugs, including immunosuppressive and prophylactic therapies for viral and fungal infections, and CKD is attributed primarily to CNI therapy^7^. The recipients of LT with the T allele of the three identified SNPs in our study may show a slower progression of renal dysfunction after the baseline immunosuppressive therapy is tapered. Notably, whereas the pre-transplant renal function did not differ between the homozygous and heterozygous groups for rs10277115 and rs792064, the CT/TT genotypic group for rs4690095 showed worse renal function prior to the LT. Our results indicate that the post-transplant decline in renal function might be independent of the pre-transplant renal function.

Based on the information about the SNPs identified in the present study, pretransplant identification of patients at a high risk for developing renal dysfunction after LT might enable providers to minimize the doses of or discontinue nephrotoxic agents, including of immunosuppressive agents, even from the early phase after LT. Since CKD after LT is caused mainly by CNIs^7^, tapering or withdrawal of CNIs and initiation of treatment with a mammalian target of rapamycin (m-TOR) inhibitor may potentially improve or slow the decline of renal function after LT^23,24^. The switch from CNI to mTOR inhibitor in the early phase after LT might alleviate deterioration of the renal function in recipients who are identified as being at a high risk for developing renal dysfunction after LT. In addition, investigation of SNPs related to drug metabolism of CNIs in the future might contribute to identification of further SNPs associated with the risk of development of renal dysfunction after LT.

In regard to the patient characteristics, the prevalences of known risk factors for the development of renal dysfunction after LT, such as age^18,25^, hypertension^18,19^, dyslipidemia^18^, and diabetes mellitus^18^, were similar between the two groups for each of the three identified SNPs in the present study. Presence of these risk factors/comorbidities did not appear to affect the differences in the renal function changes associated with the identified SNPs. To assess the renal function change over the long term after LT in the present study, we used the modified eGFR equation for the Japanese population^26^, which is independent of the muscle mass, rather than the serum creatinine, because the estimated eGFR using the Chronic Kidney Disease Epidemiology Collaboration equation has been shown to be a better predictor of the risk of CKD and may allow a better assessment of the differences in the renal function over time than that possible using serum creatinine alone in patients undergoing LT^27,28^.

This study had several limitations. First, it was a retrospective and single-center study, and the sample size was small. Second, a Japanese-specific SNP array and modified eGFR values tailored to the Japanese population were used, so that our results were ethnicity-specific. Third, for financial reasons, the Japanese-specific SNP array was used for the genotyping of SNPs only in the exploratory cohort. Fourth, many long-term survivors were included, while patients who had died prior to the start of the blood sample collection were not included, leading to a selection bias. However, this study may provide valuable information about SNPs associated with the development of renal dysfunction in the long term after LT.

In conclusion, our analysis using an ethnic-specific SNP array revealed that three SNPs, namely, rs10277115, rs4690095, and rs792064, were associated with the development of renal dysfunction in the long term after LT. Accordingly, pretransplant identification of high-risk patients for the development of renal dysfunction after LT based on the presence of these SNPs might contribute to providing personalized/precision medicine, such as individualized immunosuppression, in patients requiring lung transplantation.

## Methods

### Patients

**Figure 4:**
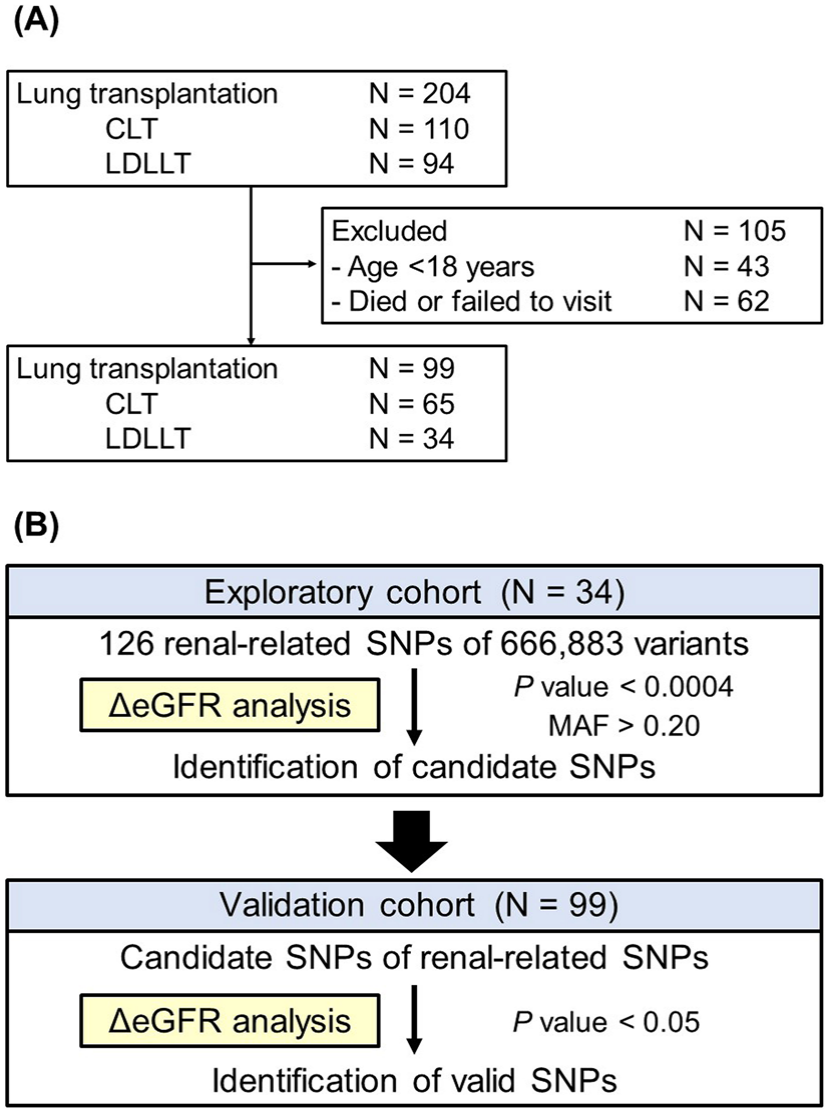
(**A**) Among 204 patients who underwent lung transplantation, including cadaveric lung transplantation (CLT) (N = 110) and living-donor lobar lung transplantation (LDLLT) (N = 94), 105 patients were excluded as they were < 18 years old (N = 43) or had died/not visited our outpatient clinic (N = 62). Blood samples were collected from the remaining 99 patients, including 65 patients who had undergone CLT and 34 patients who had undergone LDLLT. (**B**) In the exploratory cohort, 34 patients were divided into two groups according to the presence of homozygous and heterozygous combinations. The association between 126 renal-related single-nucleotide polymorphisms (SNPs) out of 666,883 variants and the postoperative changes of renal function were evaluated to explore candidate SNPs. Among SNPs with minor allele frequencies (MAF) of between 0.2 and 0.5 in the Japanese population, SNPs with *P* values of ≤ 0.0004 were identified as candidate SNPs. In the validation cohort, 99 patients were divided into two groups according to the presence of homozygous and heterozygous combinations of each candidate SNP. The associations between the candidate SNPs and the postoperative changes of renal function were evaluated. SNPs associated with renal function changes at *P* values of < 0.05 were identified as valid SNPs.

This retrospective cohort study initially included 204 patients who had undergone LT at Okayama University Hospital between October 1998 and March 2020. Among these, 43 pediatric-age patients (age < 18 years) were excluded to eliminate the effect of physical growth on the renal function; in addition, 62 patients who had died or failed to visit our outpatient clinic were also excluded. Blood samples were prospectively collected from the remaining 99 patients who were ≥ 18 years of age, including 65 patients who had undergone cadaveric LT (CLT) and 34 patients who had undergone LDLLT (Fig. 4A). No prisoner organs were procured for this study. The recipients of LT were prescribed life-long triple immunosuppressive therapy, consisting of a CNI, including tacrolimus or cyclosporine, mycophenolate mofetil or azathioprine, and a glucocorticoid. The pre-, intra-, and postoperative characteristics of the recipients were retrospectively collected from the institutional database and medical records. The Lung Allocation Score (LAS) of each patient was calculated at registration with the LT waiting list using the LAS calculator published on the OPTN website (https://optn.transplant.hrsa.gov/resources/allocation-calculators/las-calculator/) in July 2020 to assess the preoperative severity of the recipients. The severity grades of primary graft dysfunction (PGD) were assigned based on the definition of PGD proposed by the consensus report from the ISHLT^29^. CLAD was diagnosed using the classification system proposed by the ISHLT^30^.

The study protocol (No.1610-037) was approved by the institutional review board of Okayama University Hospital. Written informed consent for the study was obtained from each of the patients. All methods adopted for the study were in compliance with the relevant guidelines and regulations.

### Assessment of renal function

The estimated glomerular filtration rate (eGFR) value was used as an index of global kidney function and calculated using a revised equation for serum creatinine-based estimation of the eGFR in mL/min/1.73 m^2^ for Japanese, as follows: 194 × serum creatinine^−1.094^ × age^−0.287^ × 0.739 (if female)^26^. The preoperative eGFR values of the recipients were determined at the time of hospital admission immediately before the LT. The postoperative eGFR values of the were measured at 7 days, 1, 3, 6, and 12 months, and then every year until 10 years after the LT. Delta eGFR (ΔeGFR) was defined as the difference between the postoperative and preoperative eGFR values: ΔeGFR = postoperative eGFR value—preoperative eGFR value.

### Sample collection and preparation

Blood samples were collected from the 99 recipients for SNP genotyping between September 2016 and July 2020. The blood samples were centrifuged at 3500×*g* for 10 min, and the sedimented red blood cells were stored at − 80 °C. Genomic DNA was extracted from the buffy coat with a QIAamp DNA Mini Kit (QIAGEN K.K., Tokyo, Japan) and stored at − 80 °C.

### Genotyping of SNPs in the exploratory cohort

Among the blood samples collected from the 99 LT recipients, genomic DNA extracted from the buffy coats of only 34 samples fulfilled the criteria (genomic DNA concentration ≥ 50 ng/µL) for genotyping using a SNP array explicitly designed for the Japanese population, the Japonica array NEO. The array contains 666,883 SNPs, including 654,246 tag SNPs of autosomes and the X chromosome, and 28,298 previously identified disease-related markers. The full marker list and detailed list of disease-related SNPs are available at the jMorp website (https://jmorp.megabank.tohoku.ac.jp/downloads/#jpa). Genome-wide SNP genotyping of the samples from 34 patients were performed using this array^17^.

According to the presence of homozygous or heterozygous combinations, the 34 recipients were divided into two groups. The associations between 126 renal-related SNPs out of the 666,883 variants and the postoperative changes of renal function were evaluated to explore candidate SNPs. Among the SNPs with minor allele frequencies (MAF) of between 0.2 and 0.5 in the Japanese population, SNPs with *P* values of < 0.0004 were identified as candidate SNPs to counteract the problem of multiple comparisons (Fig. 4B).

### Genotyping of SNPs in the validation cohort

A genotype analysis for functional polymorphisms was also conducted in the samples of the remaining 65 recipients. Genomic DNA was extracted from the red blood cells with a TaqMan^®^ Sample-to-SNP™ kit (Applied Biosystems, Foster City, CA, USA). Samples were analyzed by TaqMan genotyping assay using the StepOne™ real-time polymerase chain reaction (PCR) system (Applied Biosystems) in a 96-well array plate that included 2 blank wells as negative controls. The PCR profile consisted of an initial denaturation step at 95 °C for 20 s, 40 cycles at 95 °C for 3 s, and at 60 °C for 20 s. The PCR products were analyzed using the StepOne™ Software Ver. 2.3 (Applied Biosystems). To assess the quality of the genotyping, repeat genotyping was conducted in a randomly selected 5% of the samples, and 100% agreement was confirmed.

All of the 99 recipients, consisting of the 65 recipients of the validation cohort and 34 recipients of the exploratory cohort, were divided into two groups according to the presence of homozygous or heterozygous combinations of each candidate SNP. The associations between the candidate SNPs and the postoperative changes of renal function were evaluated. SNPs with *P* values of < 0.05 were identified as valid SNPs. The same analysis was performed only in the 65 recipients except the 34 recipients of the exploratory cohort. In addition, the postoperative changes of trough level of CNIs during the first 10 years after LT were compared between the two groups for each identified SNP.

### Statistical analysis

Categorical variables were expressed as number of cases with percentages, and continuous variables were expressed in median values with ranges. Categorical variables were compared using Fisher’s exact probability test, as appropriate, and continuous variables were compared using the Mann–Whitney U test. The postoperative changes in the ΔeGFR were compared between the two groups classified according to the presence of homozygous or heterozygous combinations of each SNP using 2-way repeated measures analysis of variance (ANOVA). All statistical analyses were performed with EZR (Saitama Medical Center, Jichi Medical University, Saitama, Japan), which is a graphical user interface for R (The R Foundation for Statistical Computing, Vienna, Austria)^31^. More precisely, it is a modified version of R commander designed to add statistical functions frequently used in biostatistics. *P* < 0.05 was considered as being indicative of statistical significance.

### Supplementary Information

Supplementary Information 1.

Supplementary Information 2.

## Data Availability

The datasets generated during and/or analyzed during the current study are available from the corresponding author upon reasonable request.
