# Author Correction: Identification of genetic loci associated with renal dysfunction after lung transplantation using an ethnic-specific single-nucleotide polymorphism array

**DOI:** 10.1038/s41598-023-43995-x

**Published:** 2023-10-04

**Authors:** Yasuaki Tomioka, Seiichiro Sugimoto, Haruchika Yamamoto, Shuta Tomida, Toshio Shiotani, Shin Tanaka, Kazuhiko Shien, Ken Suzawa, Kentaroh Miyoshi, Shinji Otani, Hiromasa Yamamoto, Mikio Okazaki, Masaomi Yamane, Shinichi Toyooka

**Affiliations:** 1 Department of General Thoracic Surgery and Breast and Endocrinological Surgery, Okayama University Graduate School of Medicine, Dentistry and Pharmaceutical Sciences, Okayama, Japan; 2 Department of General Thoracic Surgery and Organ Transplant Center, Okayama University Hospital, 2‑5‑1 Shikata‑cho, Kita‑ku, Okayama, 700‑8558 Japan; 3 Center for Comprehensive Genomic Medicine, Okayama University Hospital, Okayama, Japan

Correction to: *Scientific Reports* 10.1038/s41598-023-36143-y, published online 01 June 2023

The original version of this Article contained an error.

In the Abstract, the statement

"First, eligible samples of 34 LT recipients were genotyped using the SNP array and divided into two groups, according to the presence of homozygous and heterozygous combinations of mutant alleles of the 162 renal-related SNPs.”

now reads

"First, eligible samples of 34 LT recipients were genotyped using the SNP array and divided into two groups, according to the presence of homozygous and heterozygous combinations of mutant alleles of the 126 renal-related SNPs.”

The original Article has been corrected.

