# Supplementary material for: Identification of genetic loci associated with renal dysfunction after lung transplantation using an ethnic-specific single-nucleotide polymorphism array
Source: Sci Rep. 2023 Jun 1;13:8912. doi: 10.1038/s41598-023-36143-y (PMC10235026; doi:10.1038/s41598-023-36143-y)
Supplement: Supplementary file 1 — Supplementary Information 1. [file 41598_2023_36143_MOESM1_ESM.pdf]

Supplementary Figure S1. The time course of changes of the delta estimated glomerular filtration rate (ΔeGFR) in the 65 recipients

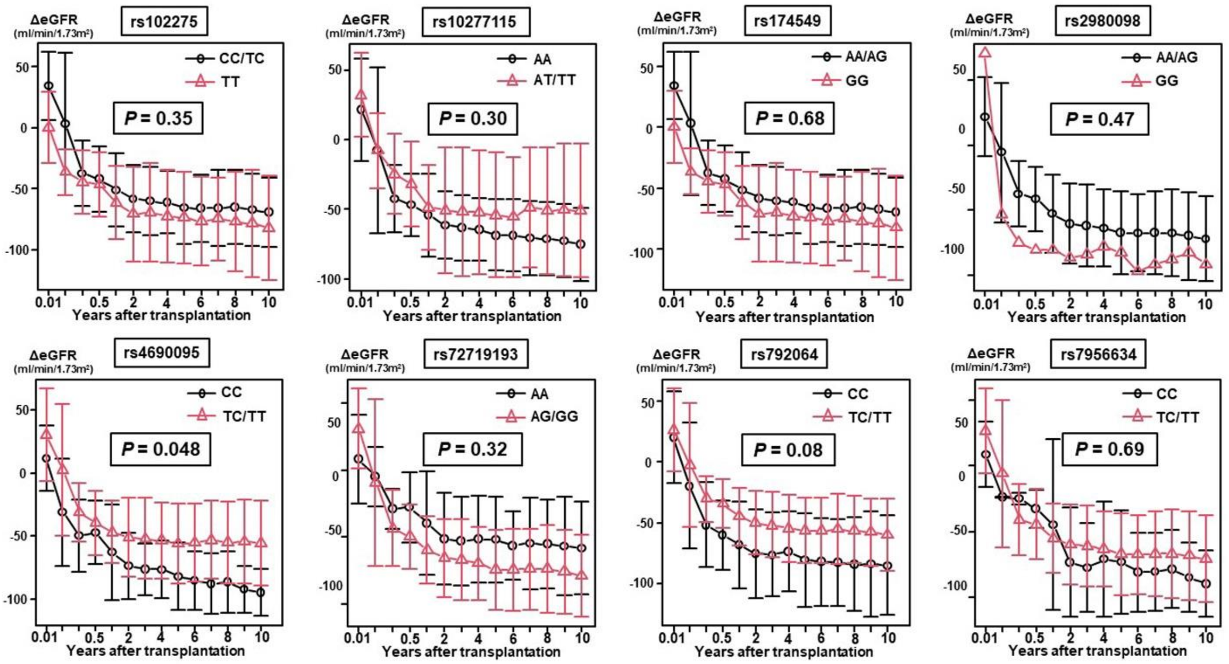

In the 65 recipients except the 34 recipients in the exploratory cohort, rs4690095 was associated with significant difference in the postoperative changes of the ΔeGFR during the first 10 years after lung transplantation (P = 0.048).
