# Supplementary material for: Identification of genetic loci associated with renal dysfunction after lung transplantation using an ethnic-specific single-nucleotide polymorphism array
Source: Sci Rep. 2023 Jun 1;13:8912. doi: 10.1038/s41598-023-36143-y (PMC10235026; doi:10.1038/s41598-023-36143-y)
Supplement: Supplementary file 2 — Supplementary Information 2. [file 41598_2023_36143_MOESM2_ESM.pdf]

Supplementary Table S1. Patient characteristics in rs10277115

Variable	AA (N = 48)	AT/TT (N = 51)	*P* value
Preoperative variables			
Age, years, median (range)	41.0 (18.0-60.0)	40.0 (21.0-68.0)	0.458
Sex (female), n (%)	31 (64.6%)	28 (54.9%)	0.413
Body mass index, kg/m2, median (range)	19.3 (10.7-25.3)	18.4 (11.0-29.3)	0.649
Diagnosis			0.034
Interstitial lung disease	13 (27.1%)	18 (35.3%)	
Emphysema	2 (4.2%)	6 (11.8%)	
Pulmonary graft-versus-host disease	3 (6.2%)	10 (19.6%)	
Lymphangioleiomyomatosis	6 (12.5%)	7 (13.7%)	
Pulmonary hypertension	8 (16.7%)	5 (9.8%)	
Bronchiectasis	9 (18.8%)	4 (7.8%)	
Other diseases	7 (14.6%)	1 (2.0%)	
Lung allocation score, median (range)	39.3 (29.8-86.0)	40.8 (22.1-89.9)	0.902
CMV mismatch (recipient negative/donor positive)	4 (8.3%)	4 (7.8%)	1
Lung donor			1
Living donor	16 (33.3%)	18 (35.3%)	
Deceased donor	32 (66.7%)	33 (64.7%)	
Calcineurin inhibitor used			0.254
Tacrolimus	39 (81.2%)	46 (90.2%)	
Cyclosporine	9 (18.8%)	5 (9.8%)	
Pretransplant creatinine (mg/dl), median (range)	0.53 (0.33-1.11)	0.62 (0.27-1.21)	0.121
Pretransplant eGFR (mL/min/1.73 m2), median (range)	107.8 (61.6-172.6)	91.3 (46.0-322.6)	0.122
Preoperative diabetes mellitus, yes	2 (4.2%)	7 (13.7%)	0.161
Preoperative hypertension, yes	2 (4.2%)	1 (2.0%)	0.61
Preoperative dyslipidemia, yes	4 (8.3%)	2 (3.9%)	0.427
Intraoperative variables			
Lung transplant procedure			
Single	8 (16.7%)	11 (21.6%)	0.614
Bilateral	40 (83.3%)	40 (78.4%)	
Operative time (min), median (range)	494.0 (219.0-730.0)	454.0 (236.0-845.0)	0.282
Ischemic time (min), median (range)	461.5 (137.0-701.0)	462.0 (82.0-787.0)	0.85
Cardiopulmonary bypass use, yes	37 (77.1%)	38 (74.5%)	0.818
Postoperative variables			
Maximum grade of PGD (0-72 h), median (range)	2 (0-3)	2 (0-3)	0.719
Acute rejection, yes	19 (39.6%)	18 (35.3%)	0.683
Anti-mediated rejection, yes	7 (14.6%)	3 (5.9%)	0.192
Postoperative diabetes mellitus, yes	5 (10.4%)	9 (17.6%)	0.391
Postoperative hypertension, yes	40 (83.3%)	41 (80.4%)	0.797
Postoperative dyslipidemia, yes	8 (16.7%)	11 (21.6%)	0.614
Hemodialysis after transplant, yes	4 (8.3%)	2 (3.9%)	0.427
Time to hemodialysis after transplant (in years), median (range)	10.9 (4.40-14.2)	14.6 (11.5-17.7)	0.533
Chronic lung allograft dysfunction, yes	12 (25.0%)	11 (21.6%)	0.813
Interval from transplant to follw-up (in years), median (range)	8.40 (0.80-23.4)	6.80 (1.80-21.9)	0.096

Data are presented as n, median (range) or n (%). CMV, cytomegalovirus; eGFR, estimated glomerular filtration rate; PGD, primary graft dysfunction

Supplementary Table S2. Patient Characteristics in rs4690095

Variable	CC (N = 20)	CT/TT (N = 79)	*P* value
Preoperative variables			
Age, years, median (range)	37.0 (22.0-60.0)	42.0 (18.0-68.0)	0.264
Sex (female), n (%)	14 (70.0%)	45 (57.0%)	0.321
Body mass index, kg/m2, median (range)	18.0 (11.0-29.3)	18.8 (10.7-29.0)	0.284
Diagnosis			0.067
Interstitial lung disease	5 (25.0%)	26 (32.9%)	
Emphysema	4 (20.0%)	4 (5.1%)	
Pulmonary graft-versus-host disease	0 (0.0%)	13 (16.5%)	
Lymphangioleiomyomatosis	5 (25.0%)	8 (10.1%)	
Pulmonary hypertension	2 (10.0%)	11 (13.9%)	
Bronchiectasis	2 (10.0%)	11 (13.9%)	
Other diseases	2 (10.0%)	6 (7.6%)	
Lung allocation score, median (range)	38.0 (32.6-71.1)	40.1 (22.1-89.9)	0.461
CMV mismatch (recipient negative/donor positive)	2 (10.0%)	6 (7.6%)	0.661
Lung donor			0.603
Living donor	8 (40.0%)	26 (32.9%)	
Deceased donor	12 (60.0%)	53 (67.1%)	
Calcineurin inhibitor used			0.15
Tacrolimus	15 (75.0%)	70 (88.6%)	
Cyclosporine	5 (25.0%)	9 (11.4%)	
Pretransplant creatinine (mg/dl), median (range)	0.51 (0.27-0.72)	0.62 (0.27-1.21)	0.023
Pretransplant eGFR (mL/min/1.73 m2), median (range)	117.8 (70.3-235.7)	94.0 (46.0-322.6)	0.008
Preoperative diabetes mellitus, yes	1 (5.0%)	8 (10.1%)	0.682
Preoperative hypertension, yes	0 (0.0%)	3 (3.8%)	1
Preoperative dyslipidemia, yes	1 (5.0%)	5 (6.3%)	1
Intraoperative variables			
Lung transplant procedure			1
Single	4 (20.0%)	15 (19.0%)	
Bilateral	16 (80.0%)	64 (81.0%)	
Operative time (min), median (range)	479.0 (238.0-785.0)	469.0 (219.0-845.0)	0.829
Ischemic time (min), median (range)	303.5 (124.0-701.0)	481.0 (82.0-787.0)	0.395
Cardiopulmonary bypass use, yes	16 (80.0%)	59 (74.7%)	0.774
Postoperative variables			
Maximum grade of PGD (0-72 h), median (range)	1.5 (0-3)	2 (0-3)	0.707
Acute rejection, yes	7 (35.0%)	30 (38.0%)	1
Anti-mediated rejection, yes	0 (0.0%)	10 (12.7%)	0.206
Postoperative diabetes mellitus, yes	3 (15.0%)	11 (13.9%)	1
Postoperative hypertension, yes	16 (80.0%)	65 (82.3%)	0.756
Postoperative dyslipidemia, yes	6 (30.0%)	13 (16.5%)	0.205
Hemodialysis after transplant, yes	2 (10.0%)	4 (5.1%)	0.598
Time to hemodialysis after transplant (in years), median (range)	12.3 (11.5-13.2)	11.4 (4.40-17.7)	1
Chronic lung allograft dysfunction, yes	2 (10.0%)	21 (26.6%)	0.146
Interval from transplant to follw-up (in years), median (range)	9.65 (1.80-21.5)	7.60 (0.80-23.4)	0.312

Data are presented as n, median (range) or n (%). CMV, cytomegalovirus; eGFR, estimated glomerular filtration rate; PGD, primary graft dysfunction

Supplementary Table S3. Patient characteristics in rs792064

Variable	CC (N = 15)	CT/TT (N = 84)	*P* value
Preoperative variables			
Age, years, median (range)	44.0 (29.0-60.0)	39.5 (18.0-68.0)	0.142
Sex (female), n (%)	6 (40.0%)	53 (63.1%)	0.151
Body mass index, kg/m2, median (range)	19.0 (12.6-23.9)	18.3 (10.7-29.3)	0.608
Diagnosis			0.993
Interstitial lung disease	4 (26.7%)	27 (32.1%)	
Emphysema	1 (6.7%)	7 (8.3%)	
Pulmonary graft-versus-host disease	2 (13.3%)	11 (13.1%)	
Lymphangioleiomyomatosis	2 (13.3%)	11 (13.1%)	
Pulmonary hypertension	3 (20.0%)	10 (11.9%)	
Bronchiectasis	2 (13.3%)	11 (13.1%)	
Other diseases	1 (6.7%)	7 (8.3%)	
Lung allocation score, median (range)	38.4 (30.2-59.2)	39.9 (22.1-89.9)	0.438
CMV mismatch (recipient negative/donor positive)	1 (6.7%)	7 (8.3%)	1
Lung donor			0.08
Living donor	2 (13.3%)	32 (38.1%)	
Deceased donor	13 (86.7%)	52 (61.9%)	
Calcineurin inhibitor used			1
Tacrolimus	13 (86.7%)	72 (85.7%)	
Cyclosporine	2 (13.3%)	12 (14.3%)	
Pretransplant creatinine (mg/dl), median (range)	0.76 (0.33-1.21)	0.56 (0.27-1.11)	0.1
Pretransplant eGFR (mL/min/1.73 m2), median (range)	84.8 (46.0-172.6)	100.9 (59.3-322.6)	0.089
Preoperative diabetes mellitus, yes	2 (13.3%)	7 (8.3%)	0.622
Preoperative hypertension, yes	0 (0.0%)	3 (3.6%)	1
Preoperative dyslipidemia, yes	1 (6.7%)	5 (6.0%)	1
Intraoperative variables			
Lung transplant procedure			0.479
Single	4 (26.7%)	15 (17.9%)	
Bilateral	11 (73.3%)	69 (82.1%)	
Operative time (min), median (range)	414.0 (219.0-613.0)	472.0 (236.0-845.0)	0.436
Ischemic time (min), median (range)	525.0 (148.0-787.0)	453.5 (82.0-774.0)	0.07
Cardiopulmonary bypass use, yes	10 (66.7%)	65 (77.4%)	0.513
Postoperative variables			
Maximum grade of PGD (0-72 h), median (range)	2 (0-3)	2 (0-3)	0.145
Acute rejection, yes	3 (20.0%)	34 (40.5%)	0.158
Anti-mediated rejection, yes	1 (6.7%)	9 (10.7%)	1
Postoperative diabetes mellitus, yes	1 (6.7%)	13 (15.5%)	0.688
Postoperative hypertension, yes	14 (93.3%)	67 (79.8%)	0.293
Postoperative dyslipidemia, yes	5 (33.3%)	14 (16.7%)	0.157
Hemodialysis after transplant, yes	1 (6.7%)	5 (6.0%)	1
Time to hemodialysis after transplant (in years), median (range)	4.4 (4.4-4.4)	13.2 (8.56-17.7)	0.333
Chronic lung allograft dysfunction, yes	3 (20.0%)	20 (23.8%)	1
Interval from transplant to follw-up (in years), median (range)	8.30 (2.30-15.2)	7.90 (0.80-23.4)	0.815

Data are presented as n, median (range) or n (%). CMV, cytomegalovirus; eGFR, estimated glomerular filtration rate; PGD, primary graft dysfunction

Supplementary Table S4. Patient characteristics of the 65 recipients in the validation cohort

Variable	N = 65
Preoperative variables	
Age, years, median (range)	40.0 (18.0-68.0)
Sex (female), n (%)	37 (56.9%)
Body mass index, kg/m2, median (range)	18.8 (7.2-29.3)
Diagnoses	
Interstitial lung disease	24 (36.9%)
Emphysema	2 (3.1%)
Pulmonary graft-versus-host disease	7 (10.8%)
Lymphangioleiomyomatosis	7 (10.8%)
Pulmonary hypertension	9 (13.8%)
Bronchiectasis	10 (15.4%)
Other diseases	6 (9.2%)
Lung allocation score, median (range)	40.3 (29.8-89.9)
CMV mismatch (recipient negative/donor positive)	6 (9.2%)
Lung donor	
Living donor	27 (41.5%)
deceased donor	38 (58.5%)
Calcineurin inhibitor	
Tacrolimus	52 (80.0%)
Cyclosporine	13 (20.0%)
Pretransplant creatinine (mg/dl), median (range)	0.57 (0.27-1.11)
Pretransplant eGFR (mL/min/1.73m2), median (range)	102.3 (59.3-322.6)
Preoperative diabetes mellitus, yes	6 (9.2%)
Preoperative hypertension, yes	2 (3.1%)
Preoperative dyslipidemia, yes	3 (4.6%)
Intraoperative variables	
Lung transplant procedure	
Single	10 (15.4%)
Bilateral	55 (84.6%)
Operative time (min), median (range)	493.5 (219.0-845.0)
Ischemic time (min), median (range)	435.0 (82.0-787.0)
Cardiopulmonary bypass use, yes	52 (80.0%)
Postoperative variables	
Maximum grade of PGD (0-72h), median (range)	2.0 (0-3)
Acute rejection, yes	27 (41.5%)
Anti-mediated rejection, yes	8 (12.3%)
Postoperative diabetes mellitus, yes	11 (16.9%)
Postoperative hypertension, yes	54 (83.1%)
Postoperative dyslipidemia, yes	10 (15.4%)
Hemodialysis after transplant, yes	4 (6.2%)
Time to hemodialysis after transplant (year), median (range)	10.9 (4.40-14.2)
Chronic lung allograft dysfunction, yes	16 (24.6%)
Time since transplant to follw-up (year), median (range)	6.39 (0.84-21.8)

Data are presented as n, median (range) or n (%). CMV, cytomegalovirus; eGFR, estimated glomerular filtration rate; PGD, primary graft dysfunction

Supplementary Table S5. Patient characteristics in rs4690095

Variable	CC (N = 12)	CT/TT (N = 53)	*P* value
Preoperative variables			
Age, years, median (range)	36.5 (22.0-60.0)	40.0 (18.0-68.0)	0.302
Sex (female), n (%)	7 (58.3%)	30 (56.6%)	1
Body mass index, kg/m2, median (range)	16.0 (11.0-29.3)	19.3 (7.16-29.0)	0.108
Diagnoses			0.237
Interstitial lung disease	5 (41.7%)	19 (35.8%)	
Emphysema	1 (8.3%)	1 (1.9%)	
Pulmonary graft-versus-host disease	0 (0.0%)	7 (13.2%)	
Lymphangioleiomyomatosis	2 (16.7%)	5 (9.4%)	
Pulmonary hypertension	2 (16.7%)	7 (13.2%)	
Bronchiectasis	0 (0.0%)	10 (18.9%)	
Other diseases	2 (10.0%)	4 (7.5%)	
Lung allocation score, median (range)	44.2 (32.9-71.1)	40.2 (29.8-89.9)	0.437
CMV mismatch (recipient negative/donor positive)	2 (16.7%)	4 (7.5%)	0.305
Lung donor			0.833
Living donor	6 (50.0%)	21 (39.6%)	
deceased donor	6 (50.0%)	32 (60.4%)	
Calcineurin inhibitor			0.053
Tacrolimus	7 (58.3%)	45 (84.9%)	
Cyclosporine	5 (41.7%)	8 (15.1%)	
Pretransplant creatinine (mg/dl), median (range)	0.51 (0.27-0.72)	0.60 (0.27-1.11)	0.214
Pretransplant eGFR (mL/min/1.73m2), median (range)	113.0 (70.3-235.7)	96.7 (59.3-322.6)	0.146
Preoperative diabetes mellitus, yes	1 (8.3%)	5 (9.4%)	1
Preoperative hypertension, yes	0 (0.0%)	8 (15.1%)	1
Preoperative dyslipidemia, yes	0 (0.0%)	3 (5.7%)	1
Intraoperative variables			
Lung transplant procedure			1
Single	2 (16.7%)	8 (15.1%)	
Bilateral	10 (83.3%)	45 (84.9%)	
Operative time (min), median (range)	504.0 (241.0-785.0)	492.0 (219.0-845.0)	0.737
Ischemic time (min), median (range)	243.5 (142.0-646.0)	460.0 (82.0-787.0)	0.483
Cardiopulmonary bypass use, yes	9 (75.0%)	43 (81.1%)	0.694
Postoperative variables			
Maximum grade of PGD (0-72h), median (range)	1.5 (0-3)	2 (0-3)	0.472
Acute rejection, yes	6 (50.0%)	21 (39.6%)	0.534
Anti-mediated rejection, yes	0 (0.0%)	8 (15.1%)	0.333
Postoperative diabetes mellitus, yes	1 (8.3%)	10 (18.9%)	0.673
Postoperative hypertension, yes	9 (75.0%)	45 (84.9%)	0.412
Postoperative dyslipidemia, yes	2 (16.7%)	8 (15.1%)	1
Hemodialysis after transplant, yes	1 (8.3%)	3 (5.7%)	0.567
Time to hemodialysis after transplant (year), median (range)	13.2 (13.2-13.2)	8.56 (4.40-14.2)	0.655
Chronic lung allograft dysfunction, yes	2 (16.7%)	14 (26.4%)	0.714
Time since transplant to follw-up (year), median (range)	7.99 (1.76-17.7)	5.94 (0.84-21.7)	0.217

Data are presented as n, median (range) or n (%). CMV, cytomegalovirus; eGFR, estimated glomerular filtration rate; PGD, primary graft dysfunction

Sample number	Lung donor	Procured institution	Procured institution address	Location, Prefecture	Location, Country
1	Living	Okayama University Hospital	2-5-1, Shikata-cho, Kita-ku, Okayama-city, Okayama, 700-8558, Japan	Okayama	Japan
2	Living	Okayama University Hospital	2-5-1, Shikata-cho, Kita-ku, Okayama-city, Okayama, 700-8558, Japan	Okayama	Japan
3	Living	Okayama University Hospital	2-5-1, Shikata-cho, Kita-ku, Okayama-city, Okayama, 700-8558, Japan	Okayama	Japan
4	Living	Okayama University Hospital	2-5-1, Shikata-cho, Kita-ku, Okayama-city, Okayama, 700-8558, Japan	Okayama	Japan
5	Living	Okayama University Hospital	2-5-1, Shikata-cho, Kita-ku, Okayama-city, Okayama, 700-8558, Japan	Okayama	Japan
6	Living	Okayama University Hospital	2-5-1, Shikata-cho, Kita-ku, Okayama-city, Okayama, 700-8558, Japan	Okayama	Japan
7	Living	Okayama University Hospital	2-5-1, Shikata-cho, Kita-ku, Okayama-city, Okayama, 700-8558, Japan	Okayama	Japan
8	Living	Okayama University Hospital	2-5-1, Shikata-cho, Kita-ku, Okayama-city, Okayama, 700-8558, Japan	Okayama	Japan
9	Living	Okayama University Hospital	2-5-1, Shikata-cho, Kita-ku, Okayama-city, Okayama, 700-8558, Japan	Okayama	Japan
10	Brain-dead	Nagoya Ekisaikai Hospital	4-66, Shonen-cho, Nakagawa Ward, Nagoya, Aichi, 454-0854, Japan	Aichi	Japan
11	Living	Okayama University Hospital	2-5-1, Shikata-cho, Kita-ku, Okayama-city, Okayama, 700-8558, Japan	Okayama	Japan
12	Living	Okayama University Hospital	2-5-1, Shikata-cho, Kita-ku, Okayama-city, Okayama, 700-8558, Japan	Okayama	Japan
13	Living	Okayama University Hospital	2-5-1, Shikata-cho, Kita-ku, Okayama-city, Okayama, 700-8558, Japan	Okayama	Japan
14	Living	Okayama University Hospital	2-5-1, Shikata-cho, Kita-ku, Okayama-city, Okayama, 700-8558, Japan	Okayama	Japan
15	Brain-dead	The Jikei University Hospital	3-19-18 Nishi-Shimbashi, Minato-ku, Tokyo 105-8471, Japan	Tokyo	Japan
16	Living	Okayama University Hospital	2-5-1, Shikata-cho, Kita-ku, Okayama-city, Okayama, 700-8558, Japan	Okayama	Japan
17	Living	Okayama University Hospital	2-5-1, Shikata-cho, Kita-ku, Okayama-city, Okayama, 700-8558, Japan	Okayama	Japan
18	Living	Okayama University Hospital	2-5-1, Shikata-cho, Kita-ku, Okayama-city, Okayama, 700-8558, Japan	Okayama	Japan
19	Living	Okayama University Hospital	2-5-1, Shikata-cho, Kita-ku, Okayama-city, Okayama, 700-8558, Japan	Okayama	Japan
20	Brain-dead	National Hospital Organization Osaka National Hospital	2-1-14 Houenzaka, Chuou-ku, Osaka, 540-0006, Japan	Osaka	Japan
21	Living	Okayama University Hospital	2-5-1, Shikata-cho, Kita-ku, Okayama-city, Okayama, 700-8558, Japan	Okayama	Japan
22	Living	Okayama University Hospital	2-5-1, Shikata-cho, Kita-ku, Okayama-city, Okayama, 700-8558, Japan	Okayama	Japan
23	Living	Okayama University Hospital	2-5-1, Shikata-cho, Kita-ku, Okayama-city, Okayama, 700-8558, Japan	Okayama	Japan
24	Living	Okayama University Hospital	2-5-1, Shikata-cho, Kita-ku, Okayama-city, Okayama, 700-8558, Japan	Okayama	Japan
25	Brain-dead	Ehime Prefectural Niihama Hospital	3-1-1, Hongo, Niihama-shi, Ehime, 792-0042, Japan	Ehime	Japan
26	Living	Okayama University Hospital	2-5-1, Shikata-cho, Kita-ku, Okayama-city, Okayama, 700-8558, Japan.	Okayama	Japan
27	Brain-dead	Fukaya Red Cross Hospital	5-8-1, Kamishibachonishi, Fukaya-city, Saitama, 366-0052, Japan	Saitama	Japan
28	Brain-dead	St. Marianna University Hospital	2-16-1, Sugao, Miyamae-ku, Kawasaki-City, Kanagawa, 216-8511, Japan	Kanagawa	Japan
29	Living	Okayama University Hospital	2-5-1, Shikata-cho, Kita-ku, Okayama-city, Okayama, 700-8558, Japan	Okayama	Japan
30	Living	Okayama University Hospital	2-5-1, Shikata-cho, Kita-ku, Okayama-city, Okayama, 700-8558, Japan	Okayama	Japan
31	Brain-dead	Chiba Emergency Medical Center	3-32-1, Isobe, Mihama-ku, Chiba-shi, Chiba, 261-0012, Japan	Chiba	Japan
32	Brain-dead	Fukushima Medical University Hospital	1, Hikarigaoka, Fukushima-city, Fukushima, 960-1295, Japan	Fukushima	Japan
33	Brain-dead	Sapporo City General Hospital	13-1-1, Kita-11-jonishi, Chuo-ku, Sapporo-city, Hokkaido, 060-8604, Japan	Hokkaido	Japan
34	Brain-dead	Kyushu University Hospital	3-1-1, Maidashi, Higashi-ku, Fukuoka-city, Fukuoka, 812-8582, Japan	Fukuoka	Japan
35	Brain-dead	University of Fukui Hospital	23-3, Matsuoka Shimoaizuki, Eiheiji-cho, Yoshida-gun, Fukui, 910-1193, Japan	Fukui	Japan
36	Brain-dead	University of Miyazaki Hospital	5200, Kihara Kiyotake, Miyazaki-city, Miyazaki, 889-1692, Japan	Miyazaki	Japan
37	Brain-dead	Shiga University of Medical Science Hospital	Seta Tsukinowa-cho, Otsu-city, Shiga, 520-2192, Japan	Shiga	Japan
38	Brain-dead	Sapporo City General Hospital	13-1-1, Kita-11-jonishi, Chuo-ku, Sapporo-city, Hokkaido, 060-8604, Japan	Hokkaido	Japan
39	Brain-dead	Tokyo Medical University Hachioji Medical Center	1163, Tatemachi, Hachioji-city, Tokyo, 193-0998, Japan	Tokyo	Japan
40	Brain-dead	St. Marianna University Hospital	2-16-1, Sugao, Miyamae-ku, Kawasaki-City, Kanagawa, 216-8511, Japan	Kanagawa	Japan
41	Brain-dead	Aichi Medical University Hospital	21, Yazako Karimata, Nagakute-cho, Aichi, 480-1131, Japan	Aichi	Japan
42	Brain-dead	Hachinohe City Hospital	1, Bishamondaira, Tamukai, Hachinohe-city, Aomori, 031-8555, Japan	Aomori	Japan
43	Brain-dead	Uwajima City Hospital	1-1, Goten-machi, Uwajima-city, Ehime, 798-8510, Japan	Ehime	Japan
44	Brain-dead	Kansai Rosai Hospital	3-1-69 Inabasou, Amagasaki-city, Hyogo, 660-8511, Japan	Hyogo	Japan
45	Brain-dead	St. Marianna University Hospital	2-16-1, Sugao, Miyamae-ku, Kawasaki-City, Kanagawa, 216-8511, Japan	Kanagawa	Japan
46	Brain-dead	Iwate Medical University Dental Center	19-1, Uchimaru, Morioka-city, Iwate, 020-0023, Japan	Iwate	Japan
47	Brain-dead	Saiseikai Utsunomiya Hospital	911-1, Takebayashi-cho, Utsunomiya-city, Tochigi, 321-0974, Japan	Tochigi	Japan
48	Brain-dead	Tokyo Medical University Hachioji Medical Center	1163, Tatemachi, Hachioji-city, Tokyo, 193-0998, Japan	Tokyo	Japan
49	Brain-dead	Yokohama Municipal Citizen's Hospital	1-1 Mitsuzawanisi-machi Kanagawa-ku, Kawasaki-city, Kanagawa, 221-0855, Japan	Kanagawa	Japan
50	Living	Okayama University Hospital	2-5-1, Shikata-cho, Kita-ku, Okayama-city, Okayama, 700-8558, Japan	Okayama	Japan
51	Brain-dead	Nakamura Memorial Hospital	14-291, Minami Ichijo Nishi, Chuo-ku, Sapporo-city, Hokkaido, 060-8570, Japan	Hokkaido	Japan
52	Brain-dead	Japan Red Cross Medical Center	4-1-22, Hiroo, Shibuya-ku, Tokyo, 150-8935, Japan	Tokyo	Japan
53	Living	Okayama University Hospital	2-5-1, Shikata-cho, Kita-ku, Okayama-city, Okayama, 700-8558, Japan	Okayama	Japan
54	Brain-dead	Saiseikai Utsunomiya Hospital	911-1, Takebayashi-cho, Utsunomiya-city, Tochigi, 321-0974, Japan	Tochigi	Japan
55	Living	Okayama University Hospital	2-5-1, Shikata-cho, Kita-ku, Okayama-city, Okayama, 700-8558, Japan	Okayama	Japan
56	Brain-dead	Tsukuba Medical Center Hospital	1-3-1 Amakubo Tsukuba-city, Ibaraki, 305-8558, Japan	Ibaraki	Japan
57	Brain-dead	Teikyo University Hosiptial	2-11-1 Kaga, Itabashi-Ku, Tokyo, 173-8606, Japan	Tokyo	Japan
58	Living	Okayama University Hospital	2-5-1, Shikata-cho, Kita-ku, Okayama-city, Okayama, 700-8558, Japan	Okayama	Japan
59	Brain-dead	Teikyo University Hosiptial	2-11-1 Kaga, Itabashi-Ku, Tokyo, 173-8606, Japan	Tokyo	Japan
60	Living	Okayama University Hospital	2-5-1, Shikata-cho, Kita-ku, Okayama-city, Okayama, 700-8558, Japan	Okayama	Japan
61	Living	Okayama University Hospital	2-5-1, Shikata-cho, Kita-ku, Okayama-city, Okayama, 700-8558, Japan	Okayama	Japan
62	Brain-dead	Japanese Red Cross Wakayama Medical Center	4-20, Komatsubara-dori, Wakayama-city, Wakayama, 640-8558, Japan	Wakayama	Japan
63	Brain-dead	Japan Red Cross Medical Center	4-1-22, Hiroo, Shibuya-ku, Tokyo, 150-8935, Japan	Tokyo	Japan
64	Living	Okayama University Hospital	2-5-1, Shikata-cho, Kita-ku, Okayama-city, Okayama, 700-8558, Japan	Okayama	Japan
64	Brain-dead	Yokohama City University Medical Center	4-57, Urafune-cho, Minami-ku, Yokohama-city, Kanagawa, 232-0024, Japan	Kanagawa	Japan
65	Brain-dead	Ise Red Cross Hospital	1-471-2, Funae, Ise-city, Mie, 516-8512, Japan	Mie	Japan
66	Brain-dead	Chiba University Hospital	1-8-1, Inohana, Chuo-ku, Chiba-city, Chiba, 260-8677, Japan	Chiba	Japan
67	Brain-dead	Okayama Red Cross Hospital	2-1-1 Aoe, Kita-ku, Okayama-city, Okayama, 700-8607, Japan	Okayama	Japan
68	Brain-dead	Kochi Health Sciences Center	2125-1 Ike, Kochi-city, Kochi, 781-8555, Japan	Kochi	Japan
69	Living	Okayama University Hospital	2-5-1, Shikata-cho, Kita-ku, Okayama-city, Okayama, 700-8558, Japan	Okayama	Japan
69	Brain-dead	Yaizu City Hospital	1000, Dobara, Yaizu-city, Shizuoka, 425-8505, Japan	Shizuoka	Japan
70	Living	Okayama University Hospital	2-5-1, Shikata-cho, Kita-ku, Okayama-city, Okayama, 700-8558, Japan	Okayama	Japan
71	Brain-dead	Hyogo Medical University Hospital	1-1, Mukogawa-cho, Nishinomiya-city, Hyogo, 663-8501, Japan	Hyogo	Japan
72	Brain-dead	Kyoto Kizugawa Hospital	26-1, Nishirokutan, Hirakawa, Joyo-city, Kyoto, 610-0101, Japan	Kyoto	Japan
73	Brain-dead	Fukuyama City Hospital	5-23-1, Zao-cho, Fukuyama-city, Hiroshima, 721-8511, Japan	Hiroshima	Japan
74	Living	Okayama University Hospital	2-5-1, Shikata-cho, Kita-ku, Okayama-city, Okayama, 700-8558, Japan	Okayama	Japan
75	Living	Okayama University Hospital	2-5-1, Shikata-cho, Kita-ku, Okayama-city, Okayama, 700-8558, Japan	Okayama	Japan
76	Brain-dead	St. Marianna University Yokohama Seibu Hospital	1197-1, Yasashi-cho, Asahi Ward, Yokohama, Kanagawa, 241-0811, Japan	Kanagawa	Japan
77	Brain-dead	The University of Tokyo Hospital	7-3-1, Hongo, Bunkyo-ku, Tokyo, 113-8655, Japan	Tokyo	Japan

78	Brain-dead	Hyogo Prefectural Nishinomiya Hospital	13-9 Rokutanji-cho, Nishinomiya-city, Hyogo, 662-0918, Japan	Hyogo	Japan
79	Brain-dead	Toyama Prefectural Central Hospital	2-2-78 Nishinage, Toyama-city, Toyama, 930-8550, Japan	Toyama	Japan
80	Brain-dead	Saiseikai Shiga Hospital	2-4-1, Ohashi, Ritto-city, Shiga, 520-3046, Japan	Shiga	Japan
81	Brain-dead	Tokyo Medical University Hachioji Medical Center	1163, Tatemachi, Hachioji-city, Tokyo, 193-0998, Japan	Tokyo	Japan
82	Brain-dead	Ota Memorial Hospital	455-1, Oshima-cho, Ota-city, Gunma, 373-8585, Japan	Gunma	Japan
83	Brain-dead	Toyohashi Municipal Hospital	50, Aza, Hachiken-nishi, Aotake-cho, Toyohashi-city, Aichi, 441-8570, Japan	Aichi	Japan
84	Brain-dead	Saiseikai Kumamoto Hospital	5-3-1 Chikami Minamiku Kumamoto-city, Kumamoto, 861-4193, Japan	Kumamoto	Japan
85	Brain-dead	Kishiwada City Hospital	1001 Gakuhara-cho, Kishiwada-city, Osaka, 596-8501, Japan	Osaka	Japan
86	Brain-dead	Kumamoto University Hospital	1-1-1, Honjo, Chuo-ku, Kumamoto-city, Kumamoto, 860-8556, Japan	Kumamoto	Japan
87	Brain-dead	Fujita Health University Hospital	1-98, Dengakugakubo, Kutsukake-cho,Toyoake-city, Aichi, 470-1192, Japan	Aichi	Japan
88	Brain-dead	Kagoshima Prefectural Oshima Hospital	18-1, Nazemanatsu-Cho, Amami-city, Kagoshima, 894-0015, Japan	Kagoshima	Japan
89	Brain-dead	Mie Prefectural General Medical Center	5450-132, Hinaga, Yokkaichi-city, Mie, 510-8561, Japan	Mie	Japan
90	Brain-dead	Kitasato University Hospital	1-15-1, Kitazato, Minami, Sagamihara-city, Kanagawa, 252-0375, Japan	Kanagawa	Japan
91	Brain-dead	Kansai Medical University Medical Center	10-15, Fumizonocho, Moriguchi-city, Osaka, 570-8507, Japan	Osaka	Japan
92	Brain-dead	Nagoya Ekisaikai Hospital	4-66, Shonen-cho, Nakagawa Ward, Nagoya, Aichi, 454-0854, Japan	Aichi	Japan
93	Brain-dead	Niigata University Medical and Dental Hospital	1-754, Asahimachi-dori, Chuo-ku, Niigata-city, Niigata, 951-8520, Japan	Niigata	Japan
94	Living	Okayama University Hospital	2-5-1, Shikata-cho, Kita-ku, Okayama-city, Okayama, 700-8558, Japan.	Okayama	Japan
95	Brain-dead	Toho University Omori Medical Center	6-11-1, Omorinishi, Ota-ku, Tokyo, 143-8541, Japan	Tokyo	Japan
96	Brain-dead	Nagaoka Red Cross Hospital	2-297-1, Senshu, Nagaoka-city, Niigata, 940-2085, Japan	Niigata	Japan
97	Brain-dead	Noto General Hospital	6-4, Fujihashimachi, Nanao-city, Ishikawa, 926-0816, Japan	Ishikawa	Japan
98	Brain-dead	Hokkaido University Hospital	Kita14, Nishi5, Kita-Ku, Sapporo-city, Hokkaido, 060-8648, Japan	Hokkaido	Japan
99	Brain-dead	Saitama Medical Center	1981, Kamoda, Kamoda, Kawagoe-city, Saitama, 350-8550, Japan	Saitama	Japan
